# Supplementary material for: Leaf Plasmodesmata Respond Differently to TMV, ToBRFV and TYLCV Infection
Source: Plants (Basel). 2021 Jul 14;10(7):1442. doi: 10.3390/plants10071442 (PMC8309360; doi:10.3390/plants10071442)
Supplement: Supplementary file 1 [file plants-10-01442-s001.zip › plants-1274517-supplementary.pdf]

Supplemental data for

Leaf plasmodesmata respond differently to TMV and TYLCV infection.

Yaarit Kutsher, Dalia Evenor, Eduard Belausov, Moshe Lapidot, Moshe Reuveni

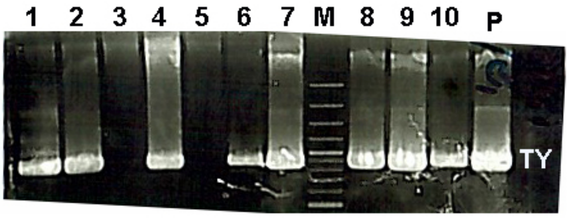

Figure S1: PCR analysis of tobacco plants infected with TYLCV. Tobacco plants that did not show any symptoms were tested for the presence of the TYLCV coat protein sequence. M = marker; P=plasmid control; lane 1-8 tobacco plant; lane 9-10 tomato plants showing symptoms of TYLCV.

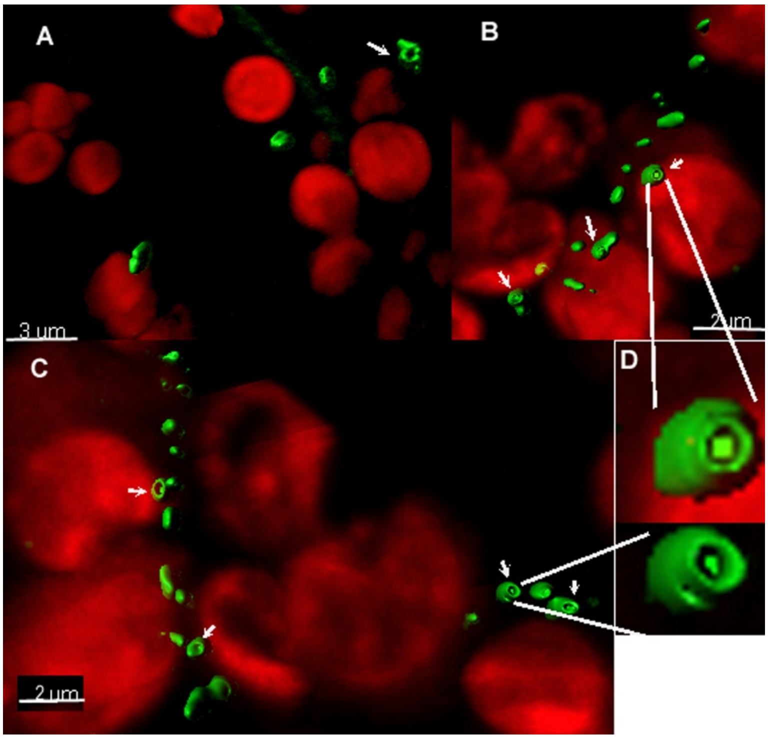

Figure S2: Imaris image of PDLP5-GFP decorated plasmodesmata. A, B, C show different cells with white arrows pointing to plasmodesmata with off-center desmotubule. D: close-up digital image of single plasmodesmata. The bar shows the distance in μm.
